# Effect of the Use of Electronic Media on the Cognitive Intelligence, Attention, and Academic Trajectory of Medical Students

**DOI:** 10.7759/cureus.79513

**Published:** 2025-02-23

**Authors:** Alejandro Hernández-Chávez, Liz Hamui-Sutton, Armando Muñoz-Comonfort, Raúl Sampieri-Cabrera

**Affiliations:** 1 Department of Physiology, Faculty of Medicine, National Autonomous University of Mexico, Mexico City, MEX; 2 Department of Medical Education, Faculty of Medicine, National Autonomous University of Mexico, Mexico City, MEX; 3 Center for Complexity Sciences, National Autonomous University of Mexico, Mexico City, MEX

**Keywords:** intelligence, learning, medical education, mental cognition, p300 potential

## Abstract

Introduction

Electronic media are an integral part of university students’ academic and personal lives; however, their excessive use may interfere with the cognitive functions critical for learning. Intelligence can be assessed through crystallized intelligence (Gc), which reflects accumulated knowledge, and fluid intelligence (Gf), which is associated with problem solving and adaptability. Also, neurophysiological measurements like the event-related potentials (ERPs), particularly P300, provide high temporal resolution measures of cognitive processing, allowing an assessment of attention (neurophysiological measures) and memory functions. This study hypothesizes that excessive screen time negatively impacts attention, fluid and crystallized intelligence, and academic performance in medical students.

Materials and methods

A total of 305 second-year medical students from a public university participated in the study (mean age: 20 years). Variables assessed included intelligence, attention, academic trajectory, and screen time. Intelligence was measured using the Shipley-2 test, and screen time was measured using a validated Screen Time Survey. For the neurophysiological measures, a subsample (n=30) underwent ERP recording with the P300 oddball paradigm for neurophysiological analysis. Academic performance was determined from standardized physiology test scores. Data were analyzed using IBM SPSS Statistics for Windows, Version 25.0 (2017; IBM Corp., Armonk, New York, United States), GraphPad Prism (Dotmatics, Boston, Massachusetts, United States), and Excel (Microsoft Corporation, Redmond, New York, United States) through descriptive and inferential statistics.

Results

A significant relationship emerged between screen time, cognitive performance, and academic achievement. Participants reported an average daily screen exposure of 7.1 ± 4.3 hours. A negative correlation was identified between screen time and standardized test scores (r = -0.24, p < 0.001), indicating that increased screen use was associated with lower academic performance. Cognitive intelligence assessments revealed that Gf (R² = 0.848) and general cognition (R² = 0.400) were significantly affected, while Gc remained stable (R² = 0.001). Neurophysiological measures showed average P300 latencies of 358.33 ± 45.6 ms and amplitudes of 259.63 ± 61.6 μV. Shorter N100 latencies (86.7 ± 24.6 ms) correlated with better academic performance (r = -0.45, R² = 0.199).

Discussion

Students with greater screen time exhibited lower standardized exam scores, suggesting that excessive digital exposure may deplete the cognitive resources essential for academic tasks. The decline in fluid and general cognitive intelligence suggests a diminished capacity for problem-solving and adaptability. Neurophysiological markers indicated prolonged latencies and reduced amplitudes of key ERPs (N100, N200, and P300), reflecting impaired attentional control and working memory in students with higher screen exposure. These findings suggest that prolonged screen use contributes to slower and less efficient cognitive processing.

Conclusions

Excessive screen time, particularly due to recreational electronic media use, negatively affects cognitive performance and academic outcomes in medical students. The observed decline appears to be mediated by deficits in attention and working memory along with a reduction in Gf, which may impair problem-solving skills and adaptability in complex academic settings.

## Introduction

Technological revolution has profoundly transformed human interactions, reshaping both educational environments and social dynamics. Today, university students regard electronic media as an indispensable tool for managing their academic and personal activities, dedicating a considerable portion of their time to their use. While these technologies facilitate access to knowledge and enrich learning, they also exert a significant effect on cognitive processes, such as attention (neurophysiological measures), problem solving, and long-term memory, which can directly impact academic performance [1-3].

Globally, individuals aged 16-64 spend an average of six hours and 40 minutes per day in front of screens, with younger populations, particularly those aged 16-24 spending up to 7.5 hours daily, primarily on smartphones, which are owned by 80% of the population. In the United States, screen time has remained stable at approximately seven hours per day since 2021 [4]. Nearly 80% of people aged 15-24 years are Internet users, and screen time has increased significantly after the COVID-19 pandemic. In Mexico, this trend is reflected in data from the Instituto Nacional de Estadística y Geografía (INEGI), which reports that individuals aged 18-24 have the highest Internet and electronic device usage, with a penetration rate of 90.5% [5]. At the Faculty of Medicine at the National Autonomous University of Mexico (UNAM), 96.7% of students from a 2019 cohort reported owning a smartphone and 71.5% a personal computer [6]. This usage pattern is reflected globally. Amez and Baert found that using smartphones for more than five hours a day correlates with increased anxiety symptoms and reduced academic performance [7]. Similarly, longitudinal studies in the United Kingdom suggest that balanced use of electronic devices fosters better academic outcomes, whereas excessive use negatively affects sustained attention [8].

The scientific literature provides evidence linking prolonged electronic device use to adverse effects on critical cognitive skills. For instance, Wacks et al. observed that excessive smartphone use impaired concentration [9]. Additionally, Jacobsen et al. found that frequent device use during lectures can reduce academic performance, while intensive social media use tends to overload the decision-making and problem-solving processes [10]. The information processing (IP) model offers a useful framework for understanding how electronic media influences cognition. This model employs a computer-mind analogy to explain how the mind receives, processes, and generates responses based on information [11]. According to the Atkinson and Shiffrin multi-store model, memory is organized into three levels: sensory memory, which is responsible for capturing signals through the senses; short-term or working memory, which has a limited capacity to handle five to seven informational elements, of which only two to four can be processed simultaneously; and long-term memory, which stores information almost indefinitely and retains elaborate cognitive schemas that support complex learning [12].

Additionally, cognitive load theory complements this perspective by describing how working memory can be affected by three types of cognitive load: (i) intrinsic load, task complexity, and prior knowledge, (ii) extraneous load, which arises when memory is used unproductively because of distractions or unclear educational materials, and (iii) germane load, which promotes learning by generating or refining prior schemas [13].

Given the increasing reliance on electronic media, it is crucial to understand its cognitive and academic implications. While prior research has linked excessive screen use to decreased attention and academic performance, the specific effects on fluid intelligence (Gf) and crystallized intelligence (Gc) remain unclear. This study hypothesizes that excessive screen time negatively impacts attention, Gf, Gc, and academic performance in medical students.

## Materials and methods

Description of the participants 

The sample was non-probabilistic and convenience-based, consisting of students enrolled in the second year of the undergraduate medical program at the Faculty of Medicine, National Autonomous University of Mexico (UNAM). Second-year medical students were selected to ensure a homogeneous sample in terms of academic background, curriculum structure, and cognitive demands. A total of 305 students participated, of whom 220 were women, 84 were men, and one was identified as non-binary. The average age of the participants was 20 years.

The protocol was approved by the Ethics and Research Committee of the Faculty of Medicine, UNAM (registration number: 049/2023). All participants signed an informed consent form and agreed to participate, including the use of their data for scientific purposes.

Measurement of the variables

The studied and analyzed variables were grouped into the following dimensions: sociodemographic, academic trajectory, intelligence, electronic media use, and neurophysiological measurements (attention) (Table 1). All participants completed an online sociodemographic survey (Appendix A) and provided information about their academic trajectory (an official document from UNAM's School Administration System) up to the time of the study.

**Table 1:** Dimensions of the study variables measured. The sociodemographic survey questionnaire is given in Appendix A. Academic Trajectory data were obtained from the educational institution. Intelligence was measured using the standardized intelligence test, Shipley-2® [14]. Electronic media use was measured by Sauce et al.'s Youth Screen Time Survey [15] (under a Creative Commons Attribution 4.0 International License), translated and modified into Spanish by the authors (Appendix B) with a Cronbach's alpha of 0.782.

Dimensions	Instruments	Type of variable
Sociodemographic	Sociodemographic survey	Age (continuous), Gender (categorical nominal), Postal Code (categorical nominal)
Academic Trajectory	Institutional academic record	Academic history (ordinal), Standardized test results (continuous/ordinal)
Intelligence	Standardized intelligence test Shipley-2®	Crystallized intelligence (Gc) (continuous), Fluid intelligence (Gf) (continuous), General cognitive intelligence (G) (continuous), Cognitive impairment (CI) (categorical ordinal)
Electronic Media Use	Screen time survey	Screen exposure time (continuous)
Neurophysiological measurements	Oddball paradigm and Event-Related Potential (ERP) recording	N100, N200, P300 (wave amplitude) (continuous)

Study Instruments

The sociodemographic survey developed by the authors (Appendix A), the standardized intelligence test, Shipley-2® [14], and Sauce et al.'s Youth Screen Time Survey [15] translated and modified into Spanish by the authors (Appendix B) were used for measurement of sociodemographic, intelligence, electronic media use dimensions, respectively.

The instruments were administered to groups of 15 students each, ensuring adequate control of the process and direct supervision by evaluators. Special care was taken to prepare the classroom environment, ensuring that it was free from visual and auditory distractions or external interference that could disrupt the participants' concentration.

Academic trajectory

This was measured by the standardized test scores for the physiology course obtained through a formal request from the Evaluation Coordination of the Department of Physiology at UNAM. The physiology course grade was chosen because it evaluates the cognitive dimensions of integration and conceptual analysis. This department provided results from three standardized tests conducted every four months, with Cronbach’s alpha values of 0.857, 0.898, and 0.907. The average of the three test scores was calculated and considered as the variable for standardized test performance.

Intelligence scale measurement

Cognitive intelligence was assessed using the Shipley-2 test [14], a psychometric tool designed to evaluate different aspects of cognitive intelligence including Gf, Gc, general intelligence, and possible signs of cognitive decline. The Shipley-2 test consists of two main sections: (i) Vocabulary scale (Gc assessment). In this section, participants selected synonyms. This section evaluates acquired knowledge such as vocabulary and verbal comprehension, which reflects prior experiences and learning. Gc is closely linked to the skills accumulated over time; (ii) Abstraction scale (Gf assessment). In this section, the participants completed pattern-related problems. This section measures logical reasoning and problem-solving abilities, which are essential aspects of fluid intelligence that are independent of prior learning and related to abstract reasoning.

The application and analysis of the test were conducted following instructions published in the original version without any modifications [14].

Screen time assessment

The screen time was assessed using the method developed by Sauce et al. [15]. The participants were asked to rate their daily use of various applications on weekdays and weekends, including activities such as watching programs and videos, playing video games, sending messages, using social media, and video chatting. The rating scale ranged from "none" to ">4 h," and means were calculated for both temporal contexts. The scale was validated in our population with a Cronbach's alpha of 0.782, supporting the internal consistency of the measurements.

Based on previous research, screen time was categorized into three main groups: (i) Watching content (programs and videos), (ii) Socializing (messages, social media, and video calls), and (iii) Playing video games.

Neurophysiological measurements (attention)

In a randomly selected subsample (n=30) of participants, electroencephalograms (EEG) were employed to record event-related potentials (ERP) to detect the P300 component. For this, the Brainbit SDK® headband device (BrainBit Inc., Rancho Santa Margarita, California, United States) was used in combination with NeuroREC™ EEG software (BrainBit Inc.). The implemented paradigm was the P300 oddball, which is characterized by the presentation of frequent and infrequent stimuli designed to elicit a differentiated brain response to rare stimuli.

The paradigm was applied using the Mentalab Explore system (Metalab GmbH, Munich, Germany) operating under an MIT (Massachusetts Institute of Technology) license [16]. This setup enabled the recording and analysis of the neurophysiological responses associated with attention and cognitive processing in a controlled and standardized environment.

Data analysis

The databases were manually constructed in Microsoft Excel (Microsoft Corporation, Redmond, Washington, United States) and analyses were conducted using IBM SPSS Statistics for Windows, Version 25.0 (2017; IBM Corp., Armonk, New York, United States), GraphPad Prism version 9 (Dotmatics, Boston, Massachusetts, United States), and Excel. The Kolmogorov-Smirnov test was used to assess normality. Descriptive statistics for the study population included measures of central tendency, dispersion, and frequency. Scatter plots and box-and-whisker plots were generated for data visualization, hypothesis testing included Pearson correlations and linear regression.

## Results

The study included 305 participants (219 female, 85 male, and one non-binary) with a mean age of 19.92 years (SD = 2.2). 

Academic trajectory

At the time of the data collection, participants had an average academic score of 7.94 (SD = 0.84) on a 0-10 grading scale. The Kolmogorov-Smirnov test (KS p>0.05) indicated that the sample data followed a normal distribution. This result justifies the application of parametric statistical analyses and the use of central-tendency measures to describe the dataset. Statistically significant differences were observed in academic performance based on prolonged use of electronic media.

Screen time

**Figure 1:**
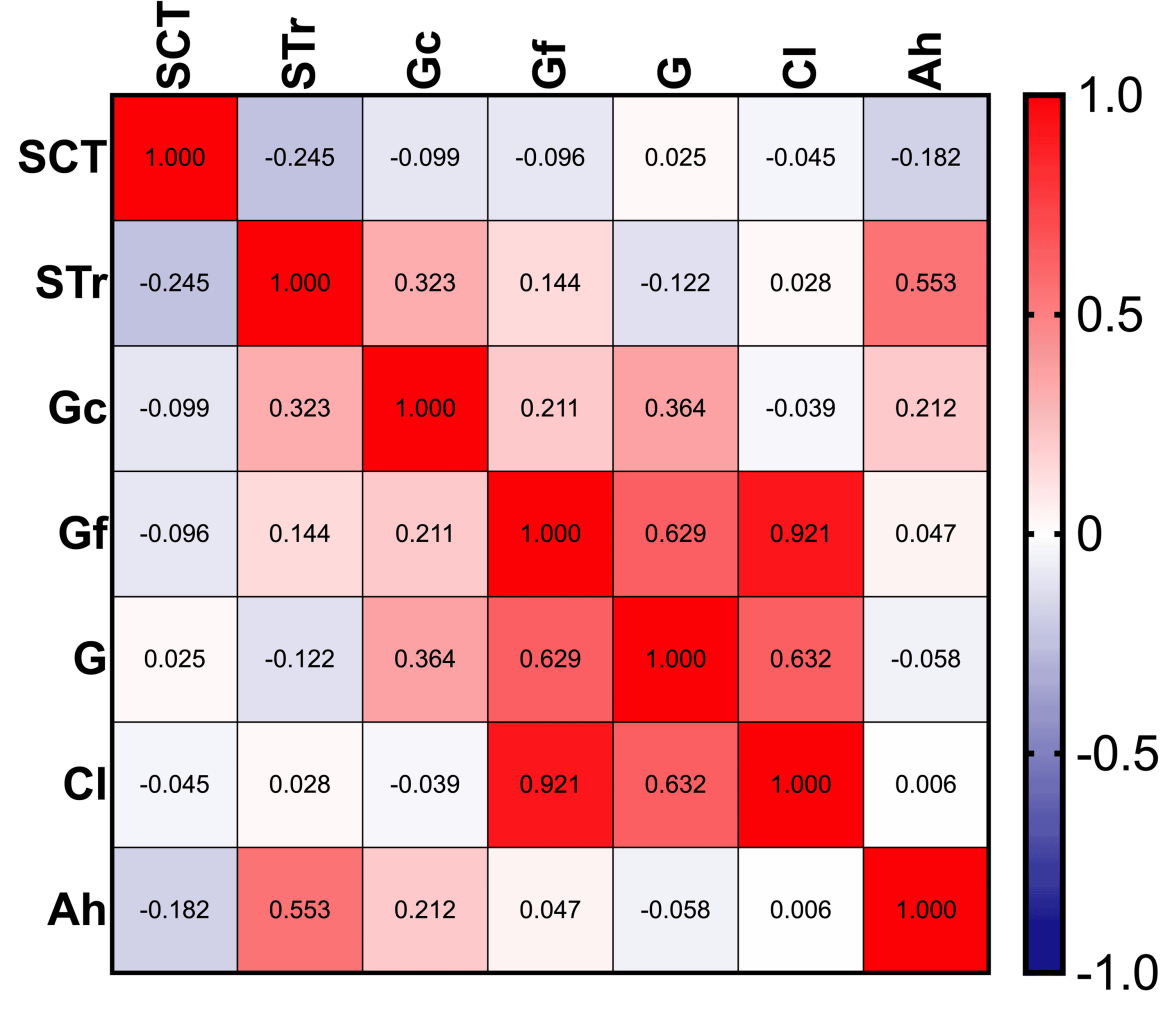
Pearson correlation matrix. This heatmap shows the correlation between the variables. SCT: screen time survey; STr: standardized test results; Gc: crystallized intelligence; Gf: fluid intelligence; G: cognitive intelligence; CI: cognitive impairment; Ah: academic history

The screen time survey reported an average total screen exposure time of 7.1 ± 4.3 hours per day. This time was divided into three main categories: 2.4 ± 1.9 hours spent on watching content (programs and videos), 4.4 ± 3.1 hours on socializing (messages, social media, and video calls), and 0.3 ± 0.7 hours on playing video games. A Pearson correlation analysis revealed a negative weak relationship between total screen time and academic performance on the standardized test (r = -0.24, p < 0.001), indicating that greater screen time was associated with lower academic performance. Figure 1 shows a correlation matrix that visually illustrates these associations.

Intelligence

**Figure 2:**
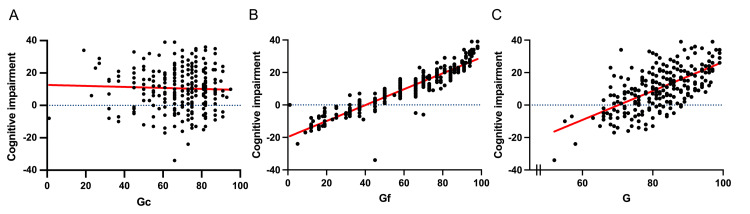
Shipley-2 intelligence test linear regression graphs for cognitive impairment and intelligence components (A) Linear regression graph of the variables cognitive impairment and crystallizes Intelligence (Gc) *R^2^*=0.001. (B) Linear regression graph of the variables cognitive impairment and fluid Intelligence (Gf) *R^2^*=0.848. (C) Linear regression graph of the variables cognitive impairment and cognitive Intelligence (G) *R^2^*=0.400.

Statistically significant differences were observed in the general cognitive intelligence scores between individuals with and without signs of cognitive impairment. The results of the Shipley-2 test showed averages of 68.7 ± 15 for Gc, 61.86 ± 23 for Gf, and 82 ± 8.8 for general cognition. Linear regression analysis revealed a significant relationship between cognitive impairment and Gf, with a coefficient of determination of R² = 0.848, as well as with general intelligence, with R² = 0.400. However, no significant relationship was identified between cognitive impairment and Gc, as evidenced by R² = 0.001. These findings suggest that cognitive impairment has a more pronounced impact on reasoning and abstract processing abilities (Gf) and global cognition (general cognitive intelligence), whereas acquired and accumulated skills (Gc) appear to remain relatively stable. Figure 2 illustrates the relationship between the components of intelligence and cognitive impairment. The cognitive impairment test results are expressed on a scale from zero upwards, with negative values indicating a probable impairment.

Electronic media use

There are significant differences in both attention and academic trajectory between students with prolonged and limited use of electronic media. The average ERP component results were as follows: (i) N100: 86.7 ± 24.6 ms, associated with early attention and initial stimulus processing; (ii) N200: 191.1 ± 31.99 ms, linked to the detection of changes in the environment; (iii) P300: 358.33 ± 45.6 ms, reflecting sustained attention and the allocation of cognitive resources; (iv) P300 amplitude: 259.63 ± 61.6 µV.

Pearson’s correlation analysis revealed a negative relationship between ERP components and departmental physiology exam grades, particularly for the N100 component (r = -0.45, R² = 0.199). This finding suggests that shorter processing times (i.e., faster latencies) are associated with better academic performance.

**Figure 3:**
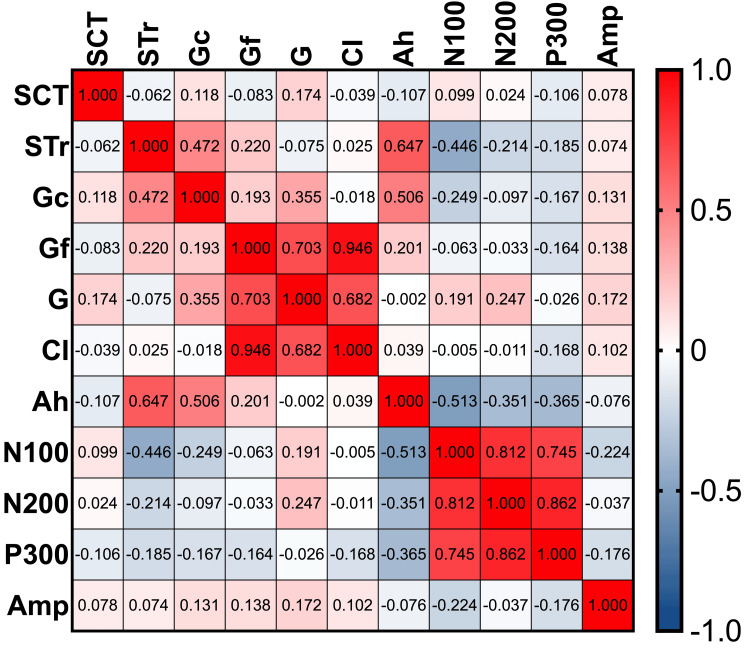
Pearson heatmap correlation matrix with P300 subsample This figure shows the correlation between the variables (n=30). SCT: screen time survey; STr: standardized test results; Gc: crystallized intelligence; Gf: fluid intelligence; G: cognitive intelligence; CI: cognitive impairment; Ah: academic history; Amp: amplitude

**Figure 4:**
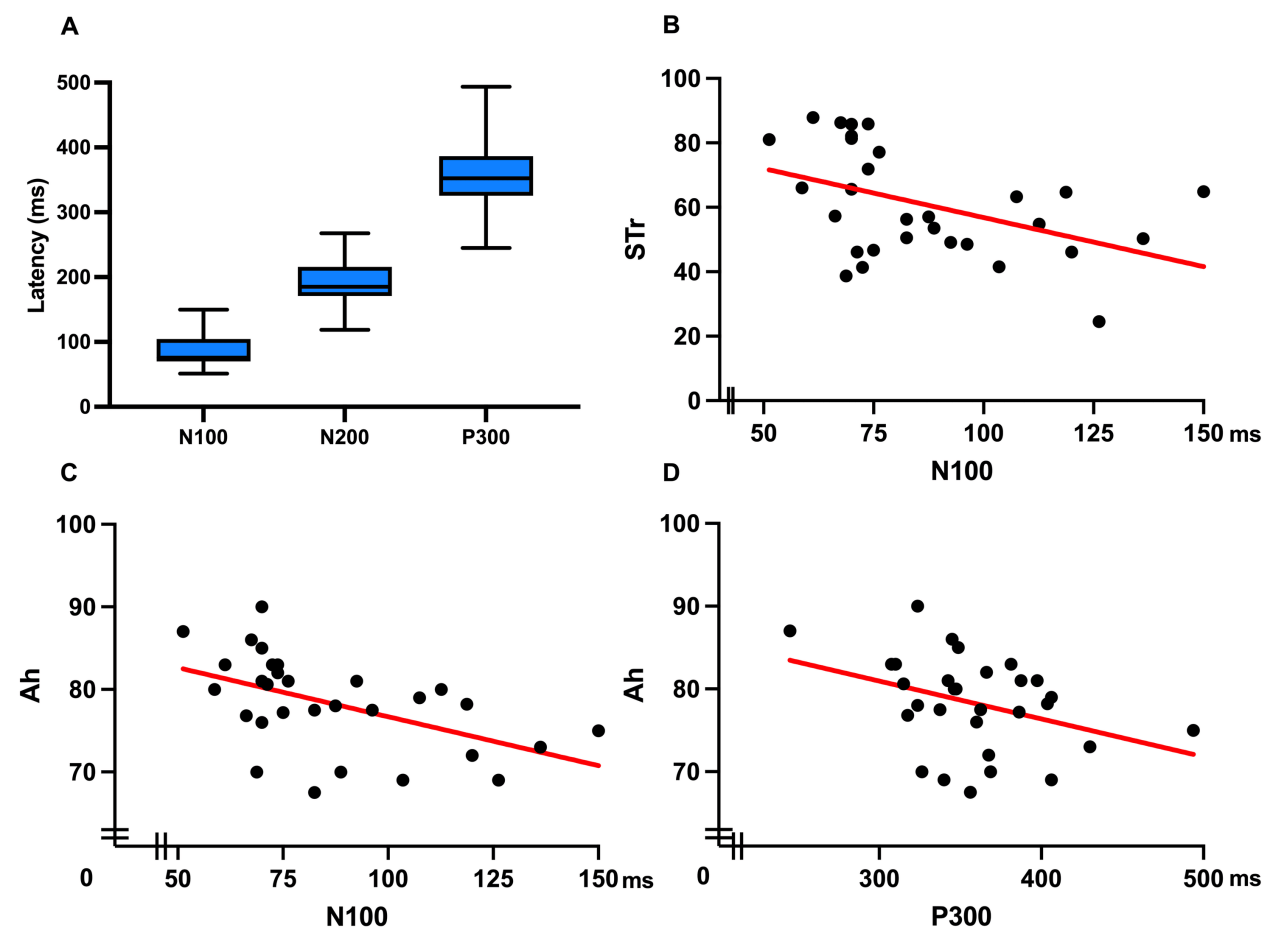
Box-and-Whiskers graph and linear regressions of the ERP sub-sample (n=30) (A) Box-and-Whiskers graph with mean and minimum-maximum range for N100 86.7± 24.6 ms (51.2-150 ms), N200 191± 31.9 (118.7-267.5 ms), P300 358.3± 45.6 ms (245-493.7 ms). (B)  Linear regression graph of STr and N100 *R^2^=0.198*. (C) Linear regression graph of Ah and N100 *R^2^=0.263*. (D) Linear regression graph of UA and P300 *R^2^=0.133*. STr: standardized test results; Ah: academic history; UA: undergraduate average

Linear regression analysis confirmed variations in the relationship between ERP components and academic performance, highlighting the complexity of these interactions. Figures 3-4 illustrate the associations between ERP components and academic grades.

## Discussion

A significant negative correlation was observed between screen time and academic performance in the physiology course. Students who spent more time using electronic media, particularly social networks, reported lower scores on standardized exams. These findings align with those of Wacks and Weinstein (2021), who demonstrated that excessive smartphone use diminishes concentration, which in turn affects learning and academic outcomes [9]. This disruption can be attributed to the diversion of cognitive resources from essential academic activities, such as problem solving and studying, to less productive tasks. Studies by Junco (2012) [17] and Kraushaar and Novak (2010) [18] have highlighted the detrimental effects of multitasking with digital devices, emphasizing that students engaging in such behaviors experience decreased task efficiency and diminished information retention.

The relationship between screen time and cognitive ability further substantiates this pattern. Using the Shipley-2 test, we identified significant differences in Gf and general cognitive intelligence between students with high and low screen time. Gf, which involves the capacity to solve novel problems and adapt to new situations, is particularly susceptible to environmental factors, such as intensive digital device use. This observation supports Cattell’s (1963) theory, which posits that, while Gc reflects accumulated knowledge and remains relatively stable, Gf is more dynamic and malleable [19]. Sauce et al. (2022) corroborate this notion by demonstrating that technologically intensive environments hinder the development of fluid intelligence [15]. These findings suggest that prolonged screen exposure might disrupt the cognitive processes that underlie problem-solving and adaptability, which are core competencies for academic success.

From a neurophysiological perspective, ERPs provide a robust framework for understanding the cognitive impact of excessive media use. Our analysis revealed prolonged latencies and reduced amplitudes of the N100, N200, and P300 waves, indicating diminished attention and working memory efficiency. These results echo those of Morand et al. (2021), who identified ERPs as reliable markers that could serve as robust assessment tools in longitudinal or clinical studies, given their moderate to very strong test-retest reliability and their potential to assess cognitive performance [20], and of Saunders and Summers (2009), who associated deficits in these waveforms with poor academic outcomes [21]. The P300 component, in particular, has been linked to processes such as stimulus evaluation and memory updating, both of which are critical for effective learning [22]. Prolonged latencies in this wave suggest slower information processing, whereas reduced amplitudes reflect diminished allocation of attentional resources, further compromising cognitive efficiency.

The impact of screen time on sleep patterns provides another avenue to explain its negative effects on academic performance. Exposure to blue light from screens during the evening hours disrupts circadian rhythms, reduces melatonin production, and impairs sleep quality [23]. Cain and Gradisar (2010) emphasized that inadequate sleep adversely affects memory consolidation, attention, and executive functions, thereby exacerbating cognitive deficits associated with excessive screen use [24]. Exelmans and Van den Bulck (2016) reported that students engaging in late-night screen time exhibited lower academic performance, a finding that is consistent with our observations [25].

Our results also underscore the importance of distinguishing between the different types of screen use. Soares et al. (2021) noted that recreational screen time, such as social media browsing, has a more pronounced negative impact on academic performance compared to academic-related screen use [26]. This distinction highlights the role of digital literacy in mitigating the adverse effects of screen exposure. McCoy (2016) advocated for fostering self-regulation skills among students to help them manage their screen time effectively, emphasizing that such interventions can improve both academic and cognitive outcomes [27].

The observed interplay between cognitive abilities and screen use can also be analyzed through the lens of dynamic cognitive theories. Sternberg (2019) argued that cognitive adaptability is intrinsically linked to environmental stimuli, suggesting that the constant influx of digital distractions might impede the development of critical thinking and problem-solving skills [28]. Schroeders et al. (2024) explored the reciprocal relationship between Gf and Gc, revealing that deficits in one domain can cascade into those in the other over time [29]. This dynamic interaction underscores the need for comprehensive interventions that address both the behavioral and cognitive dimensions of screen use.

In terms of practical implications, our findings advocate a multifaceted approach to mitigate the negative effects of excessive screen time. Educational institutions should prioritize digital literacy initiatives to promote mindful technology use. Strategies such as implementing screen time limits, encouraging device-free study periods, and incorporating evidence-based techniques such as the Pomodoro method can help students optimize their academic potential [30]. Moreover, integrating neurocognitive assessments, such as ERPs, into routine academic evaluations could enable the early identification of students at risk of cognitive decline due to excessive media use.

Finally, our research highlights the need for further longitudinal studies to elucidate the causal pathways linking screen time, cognitive ability, and academic performance. Future investigations should consider factors such as socioeconomic background, digital device accessibility, and individual differences in cognitive resilience in order to provide a more nuanced understanding of this complex relationship. By adopting a transdisciplinarity perspective that bridges psychology, neuroscience, and education, targeted interventions can be developed to foster healthier habits and enhance both learning outcomes and cognitive well-being.

Limitations

The main limitation of this study was its observational design, which prevented the establishment of causality between electronic media use and academic performance. Additionally, screen time assessment relies on self-reports, to mitigate memory bias and social desirability in the self-assessment of screen time, the instrument was validated to ensure its internal consistency. However, we recognized the lack of objective data from digital records as a limitation of this study.

The study categorized screen time into three main activities: watching content (passive), socializing (active), and playing video games (interactive). Although these general categories are useful, we recognize that a more accurate distinction between passive use (e.g., scrolling through social media) and active use (e.g., reading academic materials) would provide a more detailed understanding of their effects on cognitive performance.

Although no correlation was found between cognitive intelligence, Gf, and Gc, the time it takes students to get to school (distance time), the time they spend on recreational activities (recreational), compulsory school activities (mandatory), non-compulsory school activities (non-mandatory), and school history in previous subjects with high cognitive load, such as physiology (anatomy and biochemistry) (Appendix C), it is important to consider the role of uncontrolled factors such as socioeconomic status, pre-existing cognitive abilities, and study habits, which may influence the results. Previous research has indicated that digital literacy and sleep quality also play critical roles in academic performance [23].

## Conclusions

Prolonged exposure to electronic media is associated with lower academic performance and reduction in specific cognitive skills among medical students. A negative relationship was identified between excessive use of these devices and Gf, suggesting a decrease in adaptability and problem-solving abilities. Additionally, neurophysiological data showed alterations in ERPs, indicating reduced efficiency in attention and working memory. These effects appear to be mediated by changes in processing speed and allocation of cognitive resources, which could compromise learning and information retention.

## Figures and Tables

**Figure 5:**
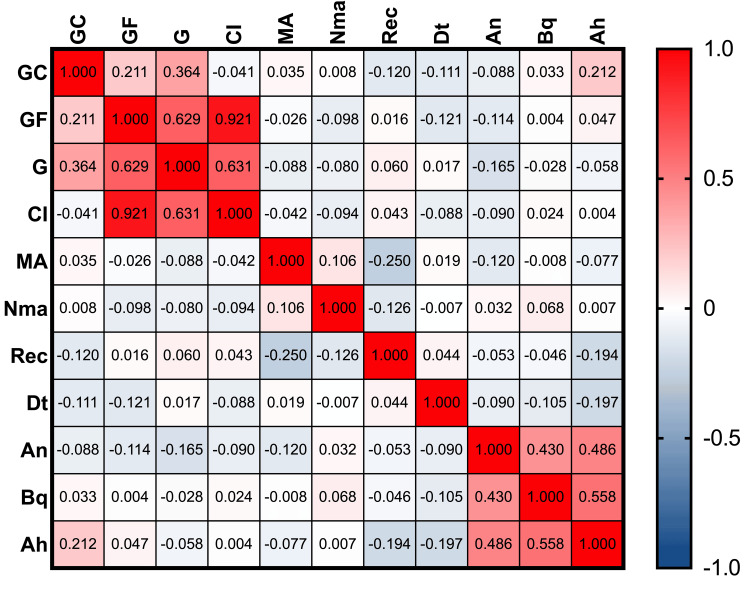
Complementary Pearson heatmap correlation matrix This figure shows the correlation between the variables (n=305). Gc: crystallized intelligence; Gf: fluid intelligence; G: cognitive intelligence; CI: cognitive impairment; MA: school daily mandatory activity time; Nma: school daily non-mandatory activity time; Rec: recreational daily time; Dt: distance daily time from home to school; An: anatomy grade note; Bq: biochemistry grade note; Ah: academic history

## Appendices

Appendix A

**Table 2:** Sociodemographic survey questionnaire

Item	Answer options (only one can be selected)
ID number	Varchar (continuous number)
Date of birth	MM/DD/YYYY
Age	Varchar
Gender	Male, Female, Nonbinary, I would rather not answer
Zip code	Varchar
On average, how long does it take you to get from home to school?	0-30 minutes, 30-60 minutes, 60-90 minutes, 120 minutes, 180 minutes or more.

Appendix B

Answer the following questions about how much screen time you spend using electronic media in a typical day.

**Table 3:** Screen time survey Reference: Sauce et al. [15]; licensed under a Creative Commons Attribution 4.0 International License (CC BY 4.0)

Item	Answer options (only one can be selected)
How much time do you watch TV, films, or series?	None, Less than 30 minutes, 1 hour, 2 hours, 3 hours, More than 4 hours.
How much time do you watch recreational videos?	None, Less than 30 minutes, 1 hour, 2 hours, 3 hours, More than 4 hours.
How much time do you watch educational videos?	None, Less than 30 minutes, 1 hour, 2 hours, 3 hours, More than 4 hours.
How much time do you play video games on a computer, console, phone, or other?	None, Less than 30 minutes, 1 hour, 2 hours, 3 hours, More than 4 hours.
How much time do you spend in sending text messages by mobile phone, tablet, or computer?	None, Less than 30 minutes, 1 hour, 2 hours, 3 hours, More than 4 hours.
How much time do you use TikTok?	None, Less than 30 minutes, 1 hour, 2 hours, 3 hours, More than 4 hours.
How much time you spend on social media apps?	None, Less than 30 minutes, 1 hour, 2 hours, 3 hours, More than 4 hours.
How much time do you use video chat or meetings?	None, Less than 30 minutes, 1 hour, 2 hours, 3 hours, More than 4 hours.

Appendix C
